# Oral oxaborole MRX-5 exhibits efficacy against pulmonary *Mycobacterium abscessus* in mouse

**DOI:** 10.1128/aac.01351-24

**Published:** 2024-10-03

**Authors:** Binayak Rimal, Ruth A. Howe, Chandra M. Panthi, Wen Wang, Gyanu Lamichhane

**Affiliations:** 1 Division of Infectious Diseases, Department of Medicine, School of Medicine, Johns Hopkins University, Baltimore, Maryland, USA; 2 MicuRx Pharmaceuticals, Inc., Foster City, California, USA; 3 Center for Nontuberculous Mycobacteria and Bronchiectasis, School of Medicine, Johns Hopkins University, Baltimore, Maryland, USA; St. George's, University of London, London, United Kingdom

**Keywords:** *Mycobacterium abscessus*, MRX-5

## Abstract

*Mycobacterium abscessus (Mab*) is an opportunistic pathogen common in patients with lung comorbidities and immunosuppression. There are no FDA-approved treatments, and current treatment has a failure rate exceeding 50%. The intravenous oxaborole MRX-6038 is active against *Mab*. This study evaluated MRX-5, the oral prodrug, against five *Mab* isolates in a mouse lung infection model. MRX-5 showed dose-dependent efficacy, with 15 and 45 mg/kg doses comparable to the standard of care, supporting progression to clinical trial.

## INTRODUCTION

Nontuberculous mycobacteria (NTM) are devastating agents of chronic pulmonary disease for those with underlying chronic lung dysfunction, such as cystic fibrosis, bronchiectasis, or chronic obstructive pulmonary disease, and for immunosuppressed patients (1). *Mycobacterium abscessus* (*Mab*), reclassified as *Mycobacteroides abscessus* (2), is one of the most commonly isolated NTMs with three subspecies (*abscessus, massiliense*, and the clinically rarer *bolletii*) causing human disease (3–5). *Mab* disease often evolves from subclinical colonization and presents as indolent, widespread disease that is challenging to eradicate. There are no FDA-approved treatments, and *Mab* isolates are often resistant to multiple antibiotics (6–8). Current standard-of-care regimens are months- or years-long multimodal courses that are expensive, logistically complex, and poorly tolerated with failure rates upward of 50% (9, 10). New treatments, especially oral options that are easier to self-administer than intravenous (IV) agents, are desperately needed.

Oxaborole antibiotics, such as epetraborole and GSK656, have been shown to be active *in vitro* and *in vivo* against *Mab* and are a promising new drug class for NTM infections (11–14). The IV formulated oxaborole MRX-6038 has comparable *in vitro* activity to epetraborole with minimum inhibitory concentration (MIC) of 0.063 to 0.25 µg/mL and MIC_90_ of 0.25 µg/mL (15, 16). A previous study evaluated the efficacy of MRX-6038 in BALB/c mouse mimicking acute *Mab* infection (15). Three days after intranasal inoculation with *Mab*, the mice were treated with 10 mg/kg of MRX-6038 administered daily by subcutaneous injection for 2 weeks, which produced approximately 3.5 log_10_ reduction in lung *Mab* burden. However, there is no study yet evaluating MRX-5, the oral prodrug of MRX-6038, or assessing either agent at extended *in vivo* time points, which are more faithful models of human *Mab* infection. Here, we evaluated the oral agent MRX-5 for the first time using a murine model of established pulmonary *Mab* infection (17) with multiple clinical *Mab* isolates.

We first determined the MICs of the active compound, MRX-6038, as well as the standard-of-care comparators clofazimine and imipenem, against a random selection of clinical *Mab* isolates (Table S1). A total of 21 *Mab* isolates recovered from bronchiectasis or cystic fibrosis patients at the Johns Hopkins Hospital and previously subspeciated by genomic analysis were considered along with the ATCC reference strain ATCC 19977 (18). MICs of MRX-6038 ranged from 0.063 to 0.63 µg/mL with MIC_50_ of 0.125 µg/mL and MIC_90_ of 0.250 µg/mL, exhibiting a broader range than prior studies (15, 16). We assessed whether an isolate with an increased MRX-6038 MIC also had increased imipenem or clofazimine MIC (Fig. S1; Table S2). The MIC data exhibited no such correlations. Based on the MRX-6038 MICs, five *Mab* isolates that represented the clinical distribution of subspecies and the full range of MRX-6038 MIC were selected to assess the efficacy of MRX-5. Four of these isolates are subspecies *abscessus* (ATCC 19977, M9501, M9507, and M9530) and one *massiliense* (M9510). M9501 and M9530 represented the most sensitive among *abscessus* isolates (MIC = 0.094 µg/mL). M9507 was the most resistant isolate overall and for the subspecies *abscessus* (MIC = 0.625 µg/mL), and M9510 was the most resistant *massiliense* (MIC = 0.250 µg/mL).

To inform MRX-5 doses for pharmacokinetic (PK) profiling, we assessed its efficacy against the laboratory reference *Mab* strain ATCC 19977 (19), with once daily oral 25, 50, and 100 mg/kg doses. Clofazimine was included as the comparator. The reduction in the lung *Mab* burdens in mice that received 25, 50, and 100 mg/kg MRX-5 was not sufficiently distinct (Fig S2; Table S3). Based on this evidence, 5, 15, and 45 mg/kg oral doses of MRX-5 were assessed in the PK study. Additionally, as all doses of MRX-5 produced a more rapid bactericidal activity than clofazimine, imipenem was included in subsequent MRX-5 assessments as imipenem exhibits bactericidal activity against *Mab* soon after its administration. Clofazimine has been noted to display delayed activity against several mycobacteria (20–22).

**Fig 1:**
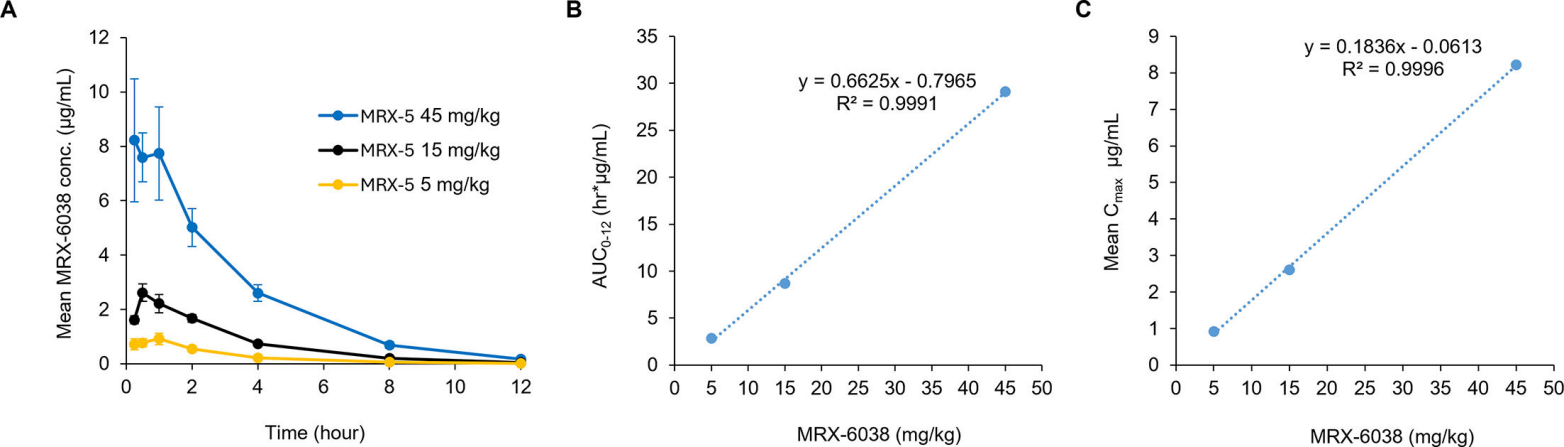
MRX-6038 pharmacokinetics profile in C3HeB/FeJ mice. (**A**) Mean (± standard deviation) of plasma concentrations of MRX-6038 vs sampling time points following oral administration of 5, 15, and 45 mg/kg bolus is shown. Dose linearity of the area under the plasma concentration vs time curve from time 0 to 12 h post dosing (AUC_0-12_) (**B**) and C_max_ (**C**), and linearity correlation coefficient are shown.

PK profiles of 5, 15, and 45 mg/kg MRX-5 were determined from plasma samples taken at intervals up to 12 hours post-oral administration (Fig. 1A; Table S4). MRX-6038 levels were measured and graphed over time. AUC_0-12_ and peak MRX-6038 concentrations showed a linear relationship with dose (Fig. 1B and C). As MRX-6038 shares its chemical class (Fig S3) and bacterial target with epetraborole, we assumed AUC/MIC would, similarly, be the key PKPD indicator for MRX-6038 against *Mab*.

**Fig 2:**
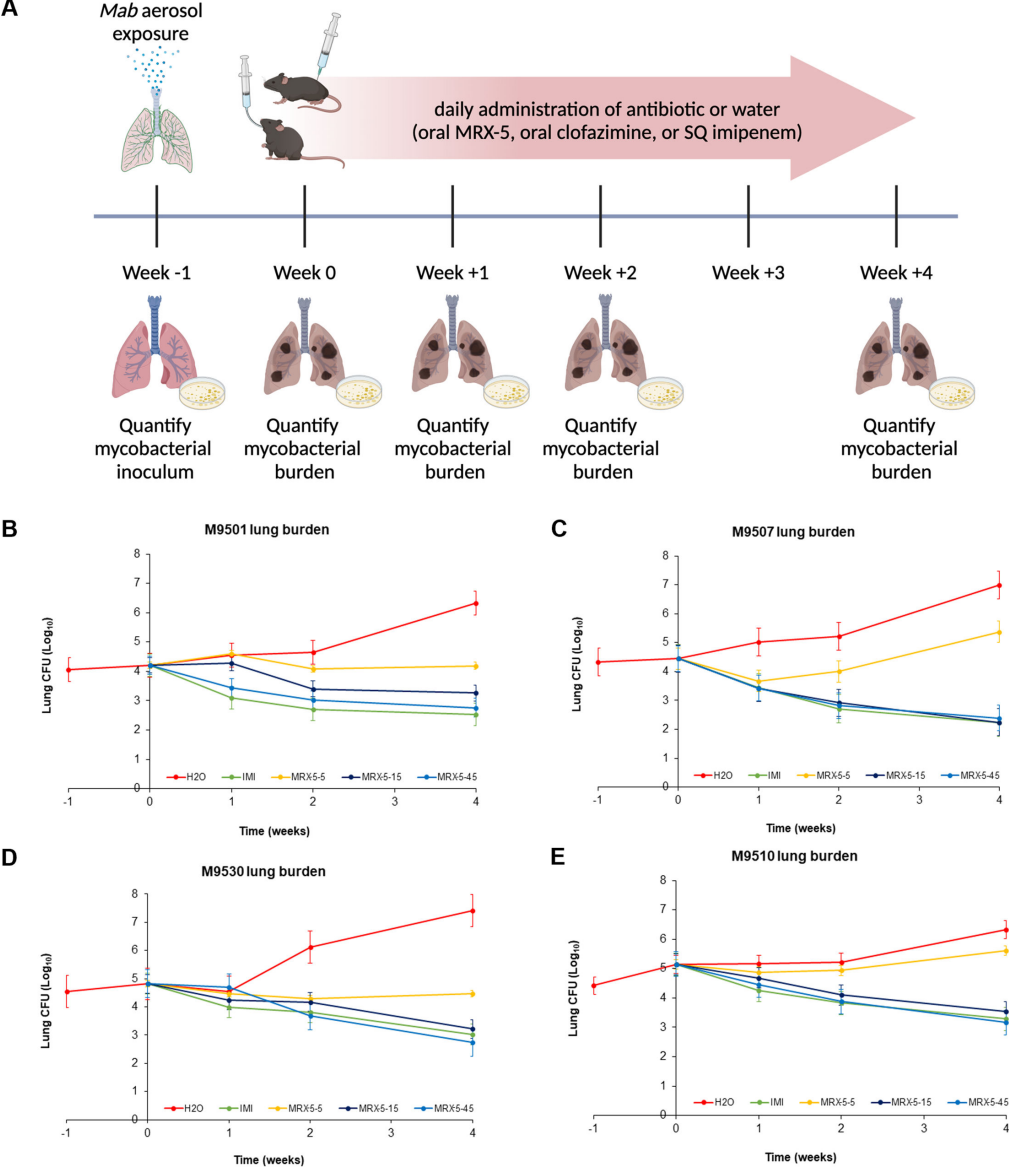
(**A**) Schematic of drug efficacy assessment in a mouse model of *Mab* lung infection. The day on which antibiotic administration was initiated is considered reference and designated “week 0.” One week prior, all mice were simultaneously infected by exposing them to aerosol of a *Mab* culture. *Mab* burden in the lungs of mice was enumerated at weeks −1, 0, +1, +2, and +4 (*n* = 5 per group at weeks −1, 0, +1, and +2, and *n* = 10 per group at week +4). *M. abscessus* burden in the lungs of mice infected with isolates M9501 (**B**), M9507 (**C**), M9530 (**D**), and M9510 (**E**). The week −1 time point corresponds to 24 h post-infection with *Mab* via aerosol. The week 0 time point marks 1 week post-infection and the beginning of treatment. The time points at weeks +1, +2, and +4 correspond to the conclusion of 1, 2, and 4 weeks of treatment, respectively, with the following regimens: once-daily sterile DI water (H_2_O), twice-daily subcutaneous imipenem at 100 mg/kg (IMI), once-daily oral MRX-5 at 5 mg/kg (MRX5-5), 15 mg/kg (MRX5-15), and 45 mg/kg (MRX5-45). The graph shows the mean *Mab* burden in the lungs along with the standard error for each group at each time point (*n* = 5 at weeks −1, 0, +1, and +2; *n* = 10 at week +4).

In subsequent studies, the efficacy of 5, 15, and 45 mg/kg MRX-5, once-daily oral, was assessed against *Mab* isolates M9501, M9507, M9530, and M9510 (Fig. 2A through E). We observed dose-dependent efficacy with isolate-specific differences in response time. As anticipated for isolates that had wide-ranging MRX-6038 MICs, we observed variable times to bactericidal response at the lower MRX-5 doses of 5 to 15 mg/kg/day, with some isolates exhibiting only a bacteriostatic response at these doses. Among the three doses, 45 mg/kg/day produced the largest reduction in the lung burdens of the isolates. The lung burdens of M9501, M9507, M9530, and M9510 gradually decreased resulting in a net reduction of 0.95, 2.20, 1.60, and 1.61 log_10_ CFU, respectively at the conclusion of 4 weeks of treatment (Fig. 2B through E). However, the net reduction produced by this dose was not statistically significant compared to the net reduction produced by 15 mg/kg dose MRX-5 or by imipenem (Table S3). Therefore, the efficacies of 15 and 45 mg/kg against the tested strains are comparable at the 4-week time point.

In summary, the MICs of MRX-6038 against isolates belonging to subspecies *abscessus* and *massiliense* were comparable, suggesting that the two subspecies have similar susceptibility to MRX-6038. The pharmacokinetics profile of MRX-5 indicated achievable exposures to effective doses with daily oral dosing. Given the heterogeneity of *Mab* isolates in the clinic, efficacy findings for a new agent are more generalizable when multiple distinct isolates are assessed (23). To meet this requirement, we tested three doses of MRX-5 against five *Mab* isolates with distinct genotypes (18) and varied susceptibility patterns to standard-of-care drugs (24). We observed variability in *Mab* lung burdens among placebo control animals and in antibiotic response at early treatment time points. These variations may be due to the genomic diversity of the isolates (18) along with some differences among the mouse cohorts. Despite using the same mouse strain, sex, and age from the same vendor, exact matching of physiological, metabolic, and immunological status is impossible. However, our study design effectively captured the clinically relevant *Mab* diversity seen in our center, and this heterogeneity underscores the need to evaluate anti-*Mab* agents over extended time points. No significant differences in *Mab* burden were seen at 1-week post-treatment for all strains nor could we fully distinguish dose efficacies at 2 weeks.

We found that MRX-5, the orally bioavailable prodrug of the novel oxaborole MRX-6038, was effective against multiple clinical *Mab* isolates in a validated mouse model of pulmonary *Mab* infection. The need for new effective, oral antibiotics targeting *Mab* continues to increase as more vulnerable patients survive transplantation, cystic fibrosis, and HIV. Our work suggests that MRX-5 could help address this clinical need and warrants further clinical investigation.

## SUPPLEMENTAL MATERIAL

Supplemental material aac.01351-24-s0001.pdf Supplemental methods, Fig. S1 to S3, and Tables S1 to S4.

ASM does not own the copyrights to Supplemental Material that may be linked to, or accessed through, an article. The authors have granted ASM a non-exclusive, world-wide license to publish the Supplemental Material files. Please contact the corresponding author directly for reuse.
