# Supplementary material for: Oral oxaborole MRX-5 exhibits efficacy against pulmonary Mycobacterium abscessus in mouse
Source: Antimicrob Agents Chemother. 2024 Oct 3;68(11):e01351-24. doi: 10.1128/aac.01351-24 (PMC11539245; doi:10.1128/aac.01351-24)
Supplement: Supplemental material — Supplemental methods, Fig. S1 to S3, and Tables S1 to S4. [file aac.01351-24-s0001.pdf]

SUPPLEMENTAL INFORMATION

Oral oxaborole MRX-5 exhibits efficacy against pulmonary *Mycobacterium abscessus* in mouse

Binayak Rimal[a], Ruth A. Howe[a], Chandra M. Panthi[a], Wen Wang[c], Gyanu Lamichhane[a,b*]

[a]Division of Infectious Diseases, Department of Medicine, School of Medicine, Johns Hopkins University, Baltimore, MD 21287, USA. [b]Center for Nontuberculous Mycobacteria and Bronchiectasis, School of Medicine, Johns Hopkins University, Baltimore, MD 21287, USA. [c]MicuRx Pharmaceuticals, Inc. Foster City, CA 94404, USA.

MATERIALS AND METHODS

Ethics statement. Animal procedures described here were performed in adherence to the national guidelines and to the Johns Hopkins University Animal Care and Use Committee.

Bacterial strains, growth media and *in vitro* growth conditions

Mab strain ATCC 19977 (1) was purchased from ATCC (Manassas, VA). *Mab* isolates M9501, M9502, M9503, M9504, M9505, M9507, M9508, M9509, M9510, M9513, M9514, M9515, M9517, M9521, M9522, M9525, M9526, M9527, M9528, M9529, M9530 and M9531 were recovered from 2005 through 2018 from patients with bronchiectasis and cystic fibrosis and obtained from the Johns Hopkins University Clinical Microbiology Laboratory (2). In a prior study, subspecies of the isolates were determined by analyzing their genome sequences, though one isolate did not meet criteria for subspecies determination (**Table S1**) (3). For MIC determination, cation-adjusted Mueller-Hinton broth (Sigma-Aldrich, catalog no. 90922) was used per CLSI guidelines (4). For infecting mice, *Mab* isolates were grown in Middlebrook 7H9 broth (Difco, catalog no. 271310) supplemented with 0.5% glycerol, 0.05% Tween-80 and 10% albumin-dextrose-sodium chloride enrichment as described (5) in an orbital shaker at 220 RPM, 37 ^{o}C. *Mab* in the mouse lungs were recovered by culturing lung homogenates on Middlebrook 7H11 selective agar (Difco, catalog # 283810) supplemented with 0.5% glycerol, 10% albumin-dextrose-sodium chloride enrichment, 50 μg/mL cycloheximide (Sigma-Aldrich, catalog # C7698), and 50 μg/mL carbenicillin (Research Products International, catalog # C46000).

Drugs

Powdered MRX-6038 and MRX-5 were provided by MicuRx pharmaceuticals. Clofazimine was purchased from Sigma-Aldrich (catalog no. C8895), imipenem was purchased from Octagon Chemicals Limited, phosphate-buffered saline (PBS), pH 7.4 from Quality Biologicals (catalog no. 114-058-101) and dexamethasone from Sigma-Aldrich (catalog no. D1756). For MIC determination: MRX-6038 was dissolved in water to prepare a stock solution of 1 mg/ml. This solution was used to prepare a fresh working stock in CAMHB for each MIC assay. Imipenem and clofazimine were dissolved in DMSO to prepare 10 mg/mL stock. This stock was used to prepare working solution in sterile deionized water. All drug solutions were filtered through 0.22 micron PVDF filter. For administration to mice: the powder MRX-5 required for each day's administration to mice was weighed at the beginning of each study into polypropylene tubes and stored at -20˚C. Each day, the aliquot allocated for the day was retrieved, then the volume of sterile deionized water necessary to prepare each dose was added and dissolved by vortex. MRX-5 solutions appear clear and transparent. Similarly, powder imipenem required for each day's administration was weighed at the beginning of each study into polypropylene tubes and stored at -20˚C. Each day, the aliquot allocated for the day was retrieved, then the volume of sterile 1x PBS, pH 7.4 necessary to prepare 12.5 mg/mL was added and dissolved by vortex followed by sonication with Sonic Dismembrator (Fisher Scientific, Model 100) at 50% power, 15 seconds per cycle, 1-2 cycles until a clear hazel solution is achieved. Clofazimine powder required for each week of administration was weighed into 50 mL polypropylene tubes and stored at -20˚C. On the first day of each week of administration, the aliquot allocated for the week was retrieved, the volume of 0.05% agarose necessary to prepare 3.125 mg/mL clofazimine was added and vortexed for 5 minutes. This suspension was sonicated at 50% power, 15 seconds per cycle, 2-3 cycles until a matte red opaque homogeneous suspension/colloid was achieved. Powder dexamethasone required for each day was measured and stored in 25-mL polypropylene tubes. Forty-two aliquots (one dose per day for 42 days) were prepared at the start of the study and stored at -

20°C. Before administration, 1x PBS (pH 7.4) necessary to prepare 1.25 mg/mL dexamethasone was added, which was then vortexed at high speed for 2 minutes. The resulting solution appeared cloudy white.

Determination of MICs

MICs of MRX-6038, imipenem and clofazimine were determined against 23 *Mab* isolates using the standard broth microdilution assay (6) with conditions specified for *Mab* in the CLSI guidelines (4). MRX-6038, imipenem and clofazimine stock solutions were diluted two-fold serially in CAMHB to generate concentrations of 8 μg/mL to 0.0156 μg/mL, 512 μg/mL to 1 μg/mL and 32 μg/mL to 0.06 μg/mL, respectively, in 200 μL final volume in each well of microtiter culture plate. For each isolate, an exponentially growing culture was used to inoculate 10^5 CFU of *Mab* into each well. Positive and negative controls included two wells with 10^5 CFU of *Mab* without any drug and two wells containing only broth. The samples were incubated at 30°C for 72 hours following CLSI guidelines. A Sensititre manual viewbox was utilized to assess *Mab* growth. The MIC was determined as the lowest antibiotic concentration at which no *Mab* growth was detected. Each MIC assay was conducted in duplicate, and the final MIC values reported represent the average of two biological and two technical replicates (**Table S1, S2**).

Determination of pharmacokinetics parameters

MRX-5 was dissolved in sterile deionized water and administered to uninfected C3HeB/FeJ mice (6 to 8 weeks old, female) by oral gavage at doses of 5-, 15-, or 45 mg/kg prepared in sterile deionized water. Five mice were used per timepoint per dose. Blood samples were collected via terminal cardiac puncture at 0.25-, 0.5-, 1-, 2-, 4-, 8-, and 12-hours post-injection, with each sample taken from a different mouse. Blood was transferred to vials with K2-EDTA and centrifuged at 5,000 g for 10 minutes to separate the plasma, which was stored at -80°C, then transferred to Meadowhawk Biolabs (Hayward, California) for the analysis. The plasma MRX-6038 concentration was determined with positive electrospray ionization using liquid chromatography/mass spectrometry API7500 systems and the PK parameters T_{max}, C_{max} and AUC 0-12

hour analyzed. An additional five mice were used to extract control plasma. These mice did not receive MRX-5.

Efficacy assessment in mice

MRX-5 efficacy was assessed against *Mab* isolates ATCC 19977, M9501, M9507, M9530 and M9510 as described in a mouse model of lung *Mab* infection (7). In this model, C3HeB/FeJ mouse strain in used as it develops defined caseous necrotic granulomas similar to that seen in humans when infected with *Mycobacterium tuberculosis.* The unique susceptibility of this mouse strain was attributed to the *sst1* locus (8). Although this mouse strain is susceptible to *M. tuberculosis*, it gradually clears lung *Mab* infection. Mild immunosuppression with dexamethasone was used to support *Mab* growth in the lungs (7). Studies with several standard-of-care antibiotics have not revealed any obvious interaction of dexamethasone with the test antibiotics (9–12). Isolates ATCC 19977, M9501, M9507, M9530 belong to the subspecies *abscessus*, while isolate M9510 belongs to the subspecies *massiliense* (3). MRX-5 efficacy was assessed against each *Mab* isolate separately. In this model, all mice in a cohort are placed in a chamber where they move freely and naturally breathe aerosol of *Mab* culture. Both negative and positive control groups were included as comparators of treatment efficacy. The positive control group received imipenem at a dosage of 100 mg/kg twice daily, subcutaneous injection, or clofazimine at a dosage of 25 mg/kg, once daily, oral, while the negative control group was given sterile deionized water, the solvent used for MRX-5. To assess the efficacy of the treatments, the *Mab* burden in the lungs of mice at the end of 1-, 2- and 4-weeks of treatment was considered endpoints. Daily refers to once per day, seven days a week.

Five cohorts of 110 C3HeB/FeJ mice per cohort, female, 5-6 weeks were procured at different times from the Jackson Laboratories for this study and acclimatized for one week prior in the holding vivarium. Identical infection and treatment protocols were used in all studies. Following the protocol of this mouse model to support *Mab* growth (7), each mouse was administered 100 μL of a 1.25 mg/mL dexamethasone

solution prepared in PBS once daily, starting one week before infection and continuing throughout the study. This dosage, equivalent to 5 mg/kg, was based on the average body weight of the mice, which was approximately 25 g at 6-8 weeks old.

To infect mice, they were housed in the chamber compartment of the Glas-Col Inhalation Exposure System (Glas-Col, Terre Haute, Indiana) and exposed simultaneously to an aerosol generated from 10 mL of *Mab* suspension at an optical density (A_{600nm}) of 0.1. This suspension was prepared by diluting a logarithmic phase culture of the *Mab* isolate in Middlebrook 7H9 broth. Typically, the *Mab* isolates reach logarithmic phase (A_{600nm} ~0.8–1.2) within 42–48 hours of incubation at 37°C with continuous shaking at 220 rpm in an orbital shaker following 1:1000 v/v inoculation. The infection process was conducted using the inhalation exposure system manufacturer's guidelines, which involved loading 10 mL *Mab* suspension into a nebulizer and executing the following automated cycles: preheating for 15 minutes, aerosol nebulization for 30 minutes, cloud decay for 30 minutes, and decontamination for 15 minutes. One day post-infection (week -1), five mice were sacrificed to assess the *Mab* colonization in their lungs. Treatment began one week after infection, with another five mice sacrificed to measure lung *Mab* burden at the start of treatment (week 0). The remaining 100 mice were randomly divided into five groups of 20. The first group, serving as the negative control, was treated orally with sterile deionized water as it was used to prepare solutions of MRX-5. The second group, positive control comparator, received either once daily oral 25 mg/kg clofazimine or twice daily, subcutaneous injection, 100 mg/kg/dose impenem. To the third, fourth and fifth group, three different doses of MRX-5 were administered orally once daily by oral gavage. In the study in which mice were infected with *Mab* ATCC 19977, 25-, 50- and 100-mg/kg MRX-5 doses were administered. In the studies in which mice were infected with isolates M9501, M9507, M9530 and M9510, 5-, 15- and 45 mg/kg MRX-5 doses were administered. Five mice per timepoint per treatment group were included at one day following infection (designated week -1), one week following infection at which is also the day on which treatment was initiated (week 0) and at the conclusion of one- and two-weeks of

treatment (week +1 and week +2). Ten mice per timepoint per treatment group were included at the conclusion of four-weeks of treatment (week +4) to provide enhanced power for the final timepoint as this timepoint is more representative of human clinical course. The lungs were extracted, homogenized, and appropriate dilutions were plated on Middlebrook 7H11 selective agar. The plates were incubated at 37°C for 5 days, after which CFU were counted.

Data analysis

Raw lung CFU data were analyzed, and the mean ± standard error was calculated for each group at each timepoint. These results were then plotted using GraphPad Prism v8.4.3. To assess the variance between treatment groups at each timepoint, a one-way ANOVA multi comparison was performed (Table S3), with significance determined at the 95% confidence level. A p-value of ≤ 0.05 was considered indicative of a non-random event, signifying significant differences in CFU burden between groups.

REFERENCES

1. Moore M, Frerichs JB. 1953. An Unusual Acid-Fast Infection of the Knee with Subcutaneous, Abscess-Like Lesions of the Gluteal Region. J Invest Dermatol 20:133–169.
2. Schwartz M, Fisher S, Story-Roller E, Lamichhane G, Parrish N. 2018. Activities of dual combinations of antibiotics against multidrug-resistant nontuberculous mycobacteria recovered from patients with cystic fibrosis. Microb Drug Resist 24:1191–1197.
3. Story-Roller E, Galanis C, Lamichhane G. 2021. β-lactam combinations that exhibit synergy against *Mycobacteroides abscessus* clinical isolates. Antimicrob Agents Chemother 65:e02545-20.
4. CLSI. 2023. Performance Standards for Susceptibility Testing of Mycobacteria, Nocardia spp., and Other Aerobic Actinomycetes, 2nd Edition. CLSI M24S2nd Editio. CLSI.
5. Larsen M. 2000. Some Common Methods in Mycobacterial Genetics, p. 313–320. *In* Hatfull, GF, Jacobs, W. R., J (eds.), Molecular Genetics of Mycobacteria. American Society for Microbiology, Washington DC.
6. Tilton RC, Lieberman L, Gerlach EH. 1973. Microdilution antibiotic susceptibility test: examination of certain variables. Appl Microbiol 26:658–65.
7. Maggioncalda EC, Story-Roller E, Mylius J, Illei P, Basaraba RJ, Lamichhane G. 2020. A mouse model of pulmonary *Mycobacteroides abscessus* infection. Nat Sci Reports 10:1–8.

8. Pichugin A V., Yan B-S, Sloutsky A, Kobzik L, Kramnik I. 2009. Dominant Role of the sst1 Locus in Pathogenesis of Necrotizing Lung Granulomas during Chronic Tuberculosis Infection and Reactivation in Genetically Resistant Hosts. Am J Pathol 174:2190–2201.

9. Story-Roller E, Maggioncalda EC, Lamichhane G. 2019. Synergistic efficacy of β-lactam combinations against *Mycobacterium abscessus* pulmonary infection in mice. Antimicrob Agents Chemother 63:e00614-19.

10. Nicklas DA, Maggioncalda EC, Story-Roller E, Eichelman B, Tabor C, Serio AW, Keepers TR, Chitra S, Lamichhane G. 2022. Potency of Omadacycline against *Mycobacteroides abscessus* Clinical Isolates In Vitro and in a Mouse Model of Pulmonary Infection. Antimicrob Agents Chemother 66:e0170421.

11. Rimal B, Nicklas DA, Panthi CM, Lippincott CK, Belz DC, Ignatius EH, Deck DH, Serio AW, Lamichhane G. 2023. Efficacy of Omadacycline-Containing Regimen in a Mouse Model of Pulmonary *Mycobacteroides abscessus* Disease. mSphere. 8(2):e0066522

12. Sriram D, Wahi R, Maggioncalda EC, Panthi CM, Lamichhane G. 2022. Clofazimine as a comparator for preclinical efficacy evaluations of experimental therapeutics against pulmonary *M. abscessus* infection in mice. Tuberculosis 137:102268.

SUPPLEMENTAL FIGURES AND TABLES

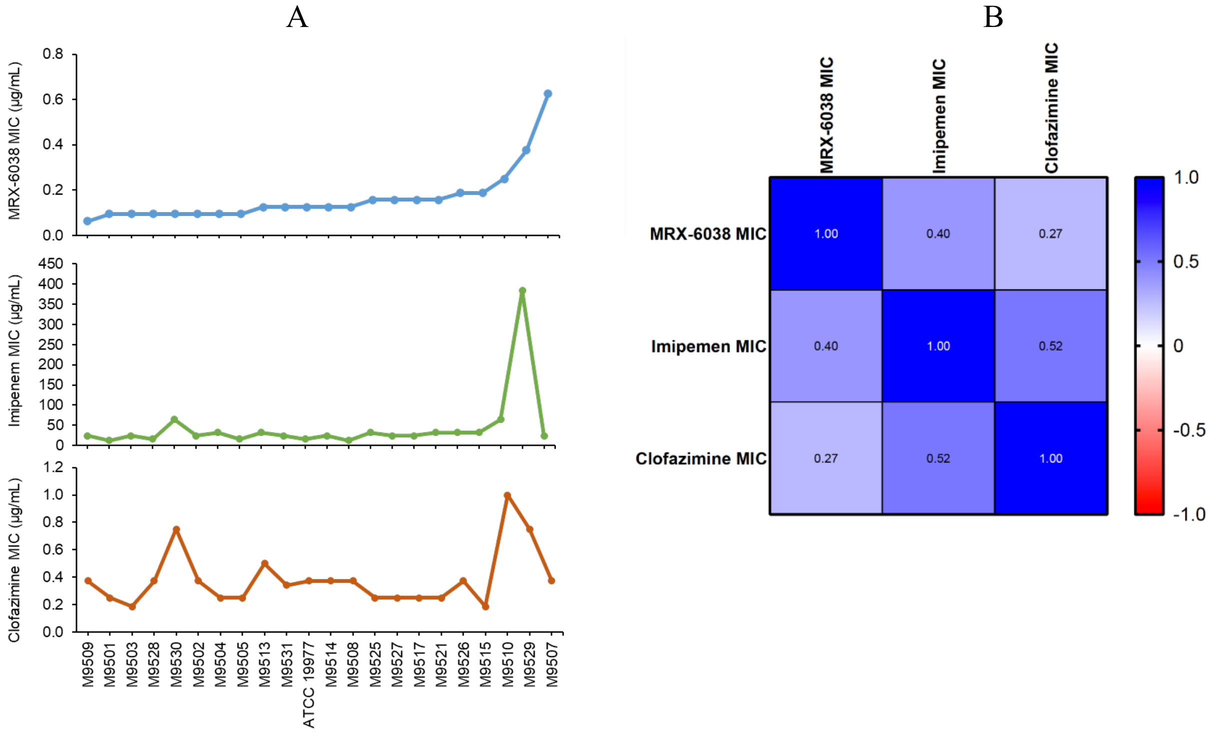

Figure S1. (**A**) MICs of MRX-6038, imipenem and clofazimine plotted against 22 different *Mab* isolates. *Mab* isolates are arranged in the order of lowest to highest MIC of MRX-6038 from left to right. (**B**) Pearson r correlation coefficients for the MICs of MRX-6038, imipenem and clofazimine for all 22 *Mab* isolates.

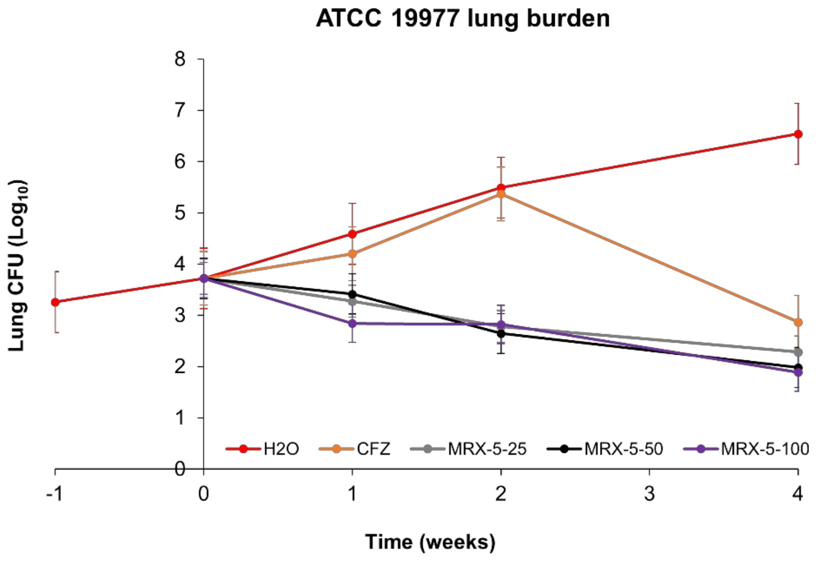

Figure S2. *M. abscessus* burden in the lungs of mice infected with isolates ATCC 19977 and treated with the following regimens: once-daily sterile DI water (H_2O), once-daily oral clofazimine at 25 mg/kg (CFZ), once-daily oral MRX-5 at 25 mg/kg (MRX5-25), 50 mg/kg (MRX5-50) and 100 mg/kg (MRX5-100). The week -1 time point corresponds to 24 hours post-infection with *Mab* via aerosol. The week 0 time point marks one week post-infection and the beginning of treatment. The time points at weeks +1, +2, and +4 correspond to the conclusion of 1, 2, and 4 weeks of treatment, respectively. The graph shows the mean *Mab* burden in the lungs along with the standard error for each group at each time point (n = 5 at weeks -1, 0, +1, and +2; n = 10 at week +4).

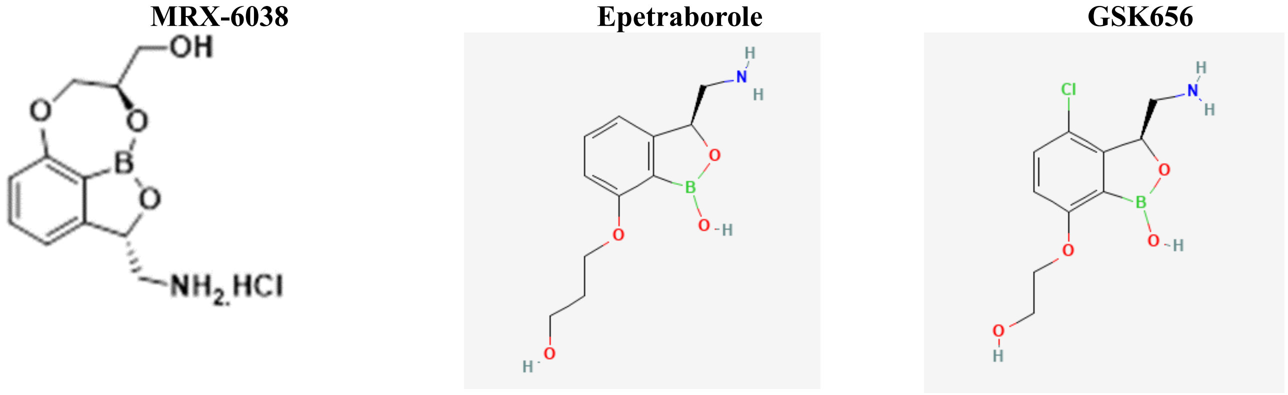

Figure S3. Chemical structures of oxaboroles MRX-6038, epetraborole and GSK656. Structures of epetraborole and GSK656 were obtained from https://pubchem.ncbi.nlm.nih.gov/compound/Epetraborole, and https://pubchem.ncbi.nlm.nih.gov/compound/Ganfeborole, respectively.

Table S1: MICs of MRX-6038, imipenem and clofazimine against *M. abscessus* isolates.

	Mab Isolate	Subspecies	Mean MIC (μg/mL)		
			MRX-6038	Imipenem	Clofazimine
	M9509	massiliense	0.063	24	0.375
	M9501	abscessus	0.094	12	0.250
	M9503	abscessus	0.094	24	0.188
	M9528	abscessus	0.094	16	0.375
	M9530	abscessus	0.094	64	0.750
	M9502	massiliense	0.094	24	0.375
	M9504	massiliense	0.094	32	0.250
	M9505	massiliense	0.094	16	0.250
	M9513	abscessus	0.125	32	0.5
	M9531	abscessus	0.125	24	0.344
MRX- 6038 MIC$_{50}$	ATCC 19977	abscessus	0.125	16	0.375
	M9514	massiliense	0.125	24	0.375
	M9508	nd	0.125	12	0.375
	M9525	abscessus	0.156	32	0.250
	M9527	abscessus	0.156	24	0.250
	M9517	massiliense	0.156	24	0.250
	M9521	massiliense	0.156	32	0.250
	M9526	abscessus	0.188	32	0.375
	M9515	massiliense	0.188	32	0.188
MRX-6038 MIC$_{90}$	M9510	massiliense	0.250	64	1.000
	M9529	abscessus	0.375	384	0.750
	M9507	abscessus	0.625	24	0.375

MICs of MRX-6038, imipenem and clofazimine were determined against 22 *M. abscessus* isolates following the Clinical and Laboratory Standards Institute guidelines. Mean MIC values were calculated from four MICs generated from two biological replicates with two technical repeats. nd: not determined.

Table S2: MIC of MRX-6038 against 22 *M. abscessus* isolates.

ISOLATES			Biological Replicate_1			Biological Replicate_2			Mean MIC (µg/mL) of two biological replicates		
			MRX 6038	IMIPENEM	CLOFAZIMINE	MRX 6038	IMIPENEM	CLOFAZIMINE	MRX 6038	IMIPENEM	CLOFAZIMINE
		T1	0.125	8	0.25	0.0625	16	0.25			
1	M9501	T2	0.125	8	0.25	0.0625	16	0.25	0.094	12	0.25
		Mean MIC*	0.125	8	0.25	0.0625	16	0.25			
		T1	0.125	32	0.125	0.0625	16	0.25			
2	M9503	T2	0.125	32	0.125	0.0625	16	0.25	0.094	24	0.19
		Mean MIC*	0.125	32	0.125	0.0625	16	0.25			
		T1	0.25	16	0.25	1	32	0.5			
3	M9507	T2	0.25	16	0.25	1	32	0.5	0.625	24	0.38
		Mean MIC*	0.25	16	0.25	1	32	0.5			
		T1	0.125	8	0.25	0.125	16	0.5			
4	M9508	T2	0.125	8	0.25	0.125	16	0.5	0.125	12	0.38
		Mean MIC*	0.125	8	0.25	0.125	16	0.5			
		T1	0.125	32	0.5	0.125	32	0.5			
5	M9513	T2	0.125	32	0.5	0.125	32	0.5	0.125	32	0.50
		Mean MIC*	0.125	32	0.5	0.125	32	0.5			
		T1	0.25	32	0.25	0.0625	32	0.25			
6	M9525	T2	0.25	32	0.25	0.0625	32	0.25	0.156	32	0.25
		Mean MIC*	0.25	32	0.25	0.0625	32	0.25			
		T1	0.25	32	0.25	0.125	32	0.5			
7	M9526	T2	0.25	32	0.25	0.125	32	0.5	0.188	32	0.38
		Mean MIC*	0.25	32	0.25	0.125	32	0.5			
		T1	0.25	32	0.25	0.0625	16	0.25			
8	M9527	T2	0.25	32	0.25	0.0625	16	0.25	0.156	24	0.25
		Mean MIC*	0.25	32	0.25	0.0625	16	0.25			
		T1	0.125	16	0.25	0.0625	16	0.5			
9	M9528	T2	0.125	16	0.25	0.0625	16	0.5	0.094	16	0.38
		Mean MIC*	0.125	16	0.25	0.0625	16	0.5			
		T1	0.25	256	0.5	0.5	512	1			
10	M9529	T2	0.25	256	0.5	0.5	512	1	0.375	384	0.75
		Mean MIC*	0.25	256	0.5	0.5	512	1			
		T1	0.125	64	0.5	0.0625	64	1			
11	M9530	T2	0.125	64	0.5	0.0625	64	1	0.094	64	0.75
		Mean MIC*	0.125	64	0.5	0.0625	64	1			
		T1	0.125	32	0.188	0.125	16	0.5			
12	M9531	T2	0.125	32	0.188	0.125	16	0.5	0.125	24	0.34
		Mean MIC*	0.125	32	0.188	0.125	16	0.5			
		T1	0.125	16	0.25	0.0625	32	0.5			
13	M9502	T2	0.125	16	0.25	0.0625	32	0.5	0.094	24	0.38
		Mean MIC*	0.125	16	0.25	0.0625	32	0.5			
		T1	0.125	32	0.25	0.0625	32	0.25			
14	M9504	T2	0.125	32	0.25	0.0625	32	0.25	0.094	32	0.25
		Mean MIC*	0.125	32	0.25	0.0625	32	0.25			
		T1	0.125	16	0.25	0.0625	16	0.25			
15	M9505	T2	0.125	16	0.25	0.0625	16	0.25	0.094	16	0.25
		Mean MIC*	0.125	16	0.25	0.0625	16	0.25			
		T1	0.0625	32	0.25	0.0625	16	0.5			
16	M9509	T2	0.0625	32	0.25	0.0625	16	0.5	0.063	24	0.38
		Mean MIC*	0.0625	32	0.25	0.0625	16	0.5			
		T1	0.25	64	1	0.25	64	1			
17	M9510	T2	0.25	64	1	0.25	64	1	0.250	64	1.00
		Mean MIC*	0.25	64	1	0.25	64	1			
		T1	0.125	32	0.25	0.125	16	0.5			
18	M9514	T2	0.125	32	0.25	0.125	16	0.5	0.125	24	0.38
		Mean MIC*	0.125	32	0.25	0.125	16	0.5			
		T1	0.25	32	0.125	0.125	32	0.25			
19	M9515	T2	0.25	32	0.125	0.125	32	0.25	0.188	32	0.19
		Mean MIC*	0.25	32	0.125	0.125	32	0.25			
		T1	0.25	32	0.25	0.0625	16	0.25			
20	M9517	T2	0.25	32	0.25	0.0625	16	0.25	0.156	24	0.25
		Mean MIC*	0.25	32	0.25	0.0625	16	0.25			
		T1	0.25	32	0.25	0.0625	32	0.25			
21	M9521	T2	0.25	32	0.25	0.0625	32	0.25	0.156	32	0.25
		Mean MIC*	0.25	32	0.25	0.0625	32	0.25			
		T1	0.125	16	0.25	0.125	16	0.5			
22	ATCC 19977	T2	0.125	16	0.25	0.125	16	0.5	0.125	16	0.38
		Mean MIC*	0.125	16	0.25	0.125	16	0.5			

Minimum inhibitory concentration (MIC) of MRX-6038 was determined against 22 *M. abscessus* isolates which included 12 that belong to the subspecies *abscessus* and 9 that belong to the subspecies *massiliense* and one whose subspecies determination is pending. MIC of MRX-6038 from two biological repeats and two technical repeats are shown. Column 10 shows the overall mean MIC of MRX-6038 calculated from four MIC values from two biological and two technical replicates shown in columns 4 and 7. Similarly, Column 11 shows overall mean MIC of imipenem calculated from four MIC values from two biological and two technical replicates shown in columns 5 and 8, and column 12 shows overall mean MIC of clofazimine calculated from four MIC values from two biological and two technical replicates shown in columns 6 and 9.

Table S3: Statistical assessment of lung *M. abscessus* burden between groups of mice receiving different treatments.

Mab Strain	Treatment Groups	*One-way ANOVA* : Multiple Comparisons (*p* values and interpretations)					
		Week +1	Interpretation of *p*-value	Week +2	Interpretation of *p*-value	Week +4	Interpretation of *p*-value
ATCC 19977	H_2O vs. CFZ	0.446	ns	0.696	ns	<0.0001	****
	H_2O vs. MRX5-25	0.017	*	<0.0001	****	<0.0001	****
	H_2O vs. MRX5-50	0.030	*	<0.0001	****	<0.0001	****
	H_2O vs. MRX5-100	0.003	**	<0.0001	****	<0.0001	****
	CFZ vs. MRX5-25	0.080	ns	<0.0001	****	0.147	ns
	CFZ vs. MRX5-50	0.131	ns	<0.0001	****	0.030	*
	CFZ vs. MRX5-100	0.015	*	<0.0001	****	0.018	*
	MRX5-25 vs. MRX5-50	0.784	ns	0.668	ns	0.446	ns
	MRX5-25 vs. MRX5-100	0.397	ns	0.888	ns	0.321	ns
	MRX5-50 vs. MRX5-100	0.267	ns	0.570	ns	0.815	ns
M9501	H_2O vs. IMI	0.040	*	0.001	**	<0.0001	****
	H_2O vs. MRX5-5	0.962	ns	0.255	ns	<0.0001	****
	H_2O vs. MRX5-15	0.677	ns	0.021	*	<0.0001	****
	H_2O vs. MRX5-45	0.105	ns	0.004	**	<0.0001	****
	IMI vs. MRX5-5	0.036	*	0.012	*	<0.0001	****
	IMI vs. MRX5-15	0.088	ns	0.171	ns	0.001	**
	IMI vs. MRX5-45	0.609	ns	0.538	ns	0.283	ns
	MRX5-5 vs. MRX5-15	0.642	ns	0.183	ns	<0.0001	****
	MRX5-5 vs. MRX5-45	0.097	ns	0.043	*	<0.0001	****
	MRX5-15 vs. MRX5-45	0.215	ns	0.434	ns	0.022	*
M9530	H_2O vs. IMI	0.056	ns	<0.0001	****	<0.0001	****
	H_2O vs. MRX5-5	0.805	ns	<0.0001	****	<0.0001	****
	H_2O vs. MRX5-15	0.268	ns	<0.0001	****	<0.0001	****
	H_2O vs. MRX5-45	0.567	ns	<0.0001	****	<0.0001	****
	IMI vs. MRX5-5	0.089	ns	0.168	ns	<0.0001	****
	IMI vs. MRX5-15	0.376	ns	0.282	ns	0.051	ns
	IMI vs. MRX5-45	0.018	*	0.723	ns	0.004	**
	MRX5-5 vs. MRX5-15	0.383	ns	0.745	ns	<0.0001	****
	MRX5-5 vs. MRX5-45	0.416	ns	0.090	ns	<0.0001	****
	MRX5-15 vs. MRX5-45	0.103	ns	0.160	ns	<0.0001	****
M9507	H_2O vs. IMI	0.001	***	0.000	***	<0.0001	****
	H_2O vs. MRX5-5	0.003	**	0.032	*	<0.0001	****
	H_2O vs. MRX5-15	0.001	***	0.000	***	<0.0001	****
	H_2O vs. MRX5-45	0.001	***	0.000	***	<0.0001	****
	IMI vs. MRX5-5	0.575	ns	0.025	*	<0.0001	****
	IMI vs. MRX5-15	0.919	ns	0.708	ns	0.983	ns
	IMI vs. MRX5-45	0.947	ns	0.841	ns	0.596	ns
	MRX5-5 vs. MRX5-15	0.509	ns	0.053	ns	<0.0001	****
	MRX5-5 vs. MRX5-45	0.532	ns	0.038	*	<0.0001	****
	MRX5-15 vs. MRX5-45	0.971	ns	0.862	ns	0.610	ns
M9510	H_2O vs. IMI	0.028	*	0.011	*	<0.0001	****
	H_2O vs. MRX5-5	0.466	ns	0.567	ns	0.054	ns
	H_2O vs. MRX5-15	0.216	ns	0.034	*	<0.0001	****
	H_2O vs. MRX5-45	0.073	ns	0.013	*	<0.0001	****
	IMI vs. MRX5-5	0.113	ns	0.036	*	<0.0001	****
	IMI vs. MRX5-15	0.272	ns	0.584	ns	0.496	ns
	IMI vs. MRX5-45	0.622	ns	0.925	ns	0.742	ns
	MRX5-5 vs. MRX5-15	0.596	ns	0.102	ns	<0.0001	****
	MRX5-5 vs. MRX5-45	0.257	ns	0.043	*	<0.0001	****
	MRX5-15 vs. MRX5-45	0.536	ns	0.649	ns	0.315	ns

Results of one-way ANOVA multi comparison of lung *M. abscessus* burdens in mice at 1-, 2- and 4-week timepoints are shown for each treatment and infection group. The mean *M. abscessus* lung burdens at 0-, 1-, 2- and 4-week timepoints following treatment are illustrated in Figures 3 and 4 of the manuscript. n=5 mice per treatment group at week +1 and +2, and n=10 mice per treatment group at week +4. Column 1 lists the unique identifier of the infecting *M. abscessus* isolates. Column 2 lists the pairs of treatment groups within the study that are compared. H_2O: sterile distilled water. CFZ: clofazimine, 25 mg/kg, once daily. IMI: imipenem, 100 mg/kg*dose, dosed twice daily. once-daily oral MRX-5 at 5 mg/kg (MRX5-5), 15 mg/kg (MRX5-15), 25 mg/kg (MRX5-25), 45 mg/kg (MRX5-45), 50 mg/kg (MRX5-50) and 100 mg/kg (MRX5-100). Columns 3, 5 and 7 list p-values of comparison of *M. abscessus* lung burdens in mice that received treatments shown in column 2. * represents p-value ≤0.05, ** represents p-value of 0.01-0.001, *** represents p-value of 0.001-0.0001, and **** represents p-value of ≤0.0001 and were interpreted as significant. "ns" represents a p-value >0.05 that was interpreted as not significant.

Table S4: MRX-6038 PK parameters in C3HeB/FeJ mice dosed orally with MRX-5

MRX-5 dose (mg/kg)	AUC_{0-12} (hr*µg/mL)	AUC/MIC*	C_{max} (µg/mL)	$T_{1/2}$ (hr)
5	2.87	11.5	0.92	1.8
15	8.67	34.7	2.61	2.0
45	29.13	116.5	8.22	2.1

AUC_{0-12}: Area under the curve up to the last timepoint
AUC/MIC*. Area under the curve up to the last timepoint divided by MIC_{90} (=0.25 µg/mL)
C_{max}: maximum concentration of MRX-6038 in plasma
$T_{1/2}$: half-life of MRX-6038 in plasma
